# Short-Circuit Fault Detection and Classification Using Empirical Wavelet Transform and Local Energy for Electric Transmission Line

**DOI:** 10.3390/s17092133

**Published:** 2017-09-16

**Authors:** Nantian Huang, Jiajin Qi, Fuqing Li, Dongfeng Yang, Guowei Cai, Guilin Huang, Jian Zheng, Zhenxin Li

**Affiliations:** 1 School of Electrical Engineering, Northeast Electric Power University, Jilin 132012, China; caigw@neepu.edu.cn; 2 Hangzhou Municipal Electric Power Supply Company of State Grid, Hangzhou 310009, China; qijiajin@126.com; 3 State Grid Ningbo Fenghua Electric Power Supply Company, Ningbo 315500, China; lfq303@126.com; 4 Jiangxi Economic and Technical Research Institute of State Grid, Nanchang 330043, China; hgl666666@163.com (G.H.); acmilan2290@126.com (J.Z.); 5 Jilin Electric Power Supply Company of State Grid, Jilin 132012, China; lizhenxinwyy@126.com

**Keywords:** short-circuit fault, empirical wavelet transform, local energy, support vector machine

## Abstract

In order to improve the classification accuracy of recognizing short-circuit faults in electric transmission lines, a novel detection and diagnosis method based on empirical wavelet transform (EWT) and local energy (LE) is proposed. First, EWT is used to deal with the original short-circuit fault signals from photoelectric voltage transformers, before the amplitude modulated-frequency modulated (AM-FM) mode with a compactly supported Fourier spectrum is extracted. Subsequently, the fault occurrence time is detected according to the modulus maxima of intrinsic mode function (IMF_2_) from three-phase voltage signals processed by EWT. After this process, the feature vectors are constructed by calculating the LE of the fundamental frequency based on the three-phase voltage signals of one period after the fault occurred. Finally, the classifier based on support vector machine (SVM) which was constructed with the LE feature vectors is used to classify 10 types of short-circuit fault signals. Compared with complementary ensemble empirical mode decomposition with adaptive noise (CEEMDAN) and improved CEEMDAN methods, the new method using EWT has a better ability to present the frequency in time. The difference in the characteristics of the energy distribution in the time domain between different types of short-circuit faults can be presented by the feature vectors of LE. Together, simulation and real signals experiment demonstrate the validity and effectiveness of the new approach.

## 1. Introduction

The detection and classification of short-circuit faults in power transmission lines are the basis for accurately judging the fault phase. The accurate removal of the fault phase reduces the further negative impact of the failure in the power system. It is very useful for enhancing the stability of the power system, boosting the transient stability of the system, and improving the quality of the power supply [1,2].

Having a method to accurately and efficiently classify short-circuit faults is the basis of fault clearance for power transmission lines. Fault voltage signals achieved from the sensors of photoelectric voltage transformers contain various transient components due to uncertain factors, such as fault location, fault time, and transition resistance of transmission lines. It is useful but complex to analyze and recognize these fault voltage signals. Generally, classification of short-circuit faults based on the voltage signals include three steps: signal processing, feature extraction, and pattern recognition.

Signal processing is the basis of classifying short-circuit faults. The time-frequency analysis approach is commonly used in the processing of short-circuit fault signals, which mainly includes the wavelet transform (WT) [3,4]; wavelet packet transform (WPT) [5,6]; S-transform (ST) [7,8]; empirical mode decomposition (EMD) [9]; ensemble empirical mode decomposition (EEMD) [10,11]; ensemble empirical mode decomposition (EEMD); complete ensemble empirical mode decomposition with adaptive noise (CEEMDAN) [12]; and improved complete ensemble empirical mode decomposition with adaptive noise (Improved CEEMDAN) [13]. WT and WPT are more capable of analyzing the time frequency of signals. However, they still have limitations such as being easily influenced by noise, limited frequency resolution in high-frequency parts as well as difficulty in selecting the mother wavelet function and decomposition scale [1,14]. ST has good time-frequency resolution and noise immunity, but ST also has a very high computation complexity. Therefore, it is difficult to analyze fault signals at a high sampling rate. Compared with the WT, WPT, and ST methods, the EMD method has advanced adaptability. As it can adaptively decompose the non-linear and non-stationary signals into several intrinsic mode functions (IMFs), which reflect components at different frequencies. It is considerably more convenient to extract the features of fault signals from IMFs. However, EMD has some limitations due to mode mixing, pseudomode, and so on. The EEMD method has a higher time-frequency resolution than the EMD and retains the problem of mode mixing. Furthermore, the reconstructed signal and the final trend of EEMD contains residual noise, while different realizations of the signal and noise generates different numbers of modes. The CEEMDAN method proposed in a previous reference [12] was proved to be a significant improvement of EEMD, which realizes a small reconstruction error and solves the problem of generating a different number of modes by adding different noise to signals. However, the CEEMDAN method still has some problems, such as residual noise contained in its modes. In order to solve these problems of CEEMDAN, the improved CEEMDAN method was proposed by reference [13]. The empirical wavelet transform (EWT) is a new adaptive signal processing method [15], which combines the adaptability of EMD with a wavelet decomposition framework. Compared to EMD and its improved methods, EWT has the following advantages:

- EWT decomposes the signal spectrum adaptively, and constructs orthogonal wavelet filter banks to extract amplitude modulated-frequency modulated (AM-FM) components with a compactly supported Fourier spectrum. Therefore, it can accurately decompose the short-circuit fault signals into IMFs to avoid mode mixing.
- The EWT approach has been proven by the classical wavelet theory. However, the orthogonality of IMFs obtained by the EMD method has not been shown in previous studies.
- IMFs obtained by the EMD method require many iterations and numerous calculations. However, EWT requires less calculations to obtain the IMFs from fault signals based on the wavelet method.

Therefore, EWT is more suitable for analyzing short-circuit fault signals with non-stationary characteristics.

The features used directly affect the classification of short-circuit faults. The features of short-circuit fault signals can be extracted from the time-frequency matrix of short-circuit faults. In the present study, various entropies such as Shannon entropy [16], Shannon energy entropy (EE) [17], Shannon energy spectrum entropy [18], Shannon time entropy (TE) [5], and Shannon singular entropy (SE) [19] have been used to characterize the time-frequency characteristics of short-circuit faults. When short-circuit fault signals are described by entropy, it is difficult to satisfy the requirement of different sampling rates by a uniform standard of selecting sliding window parameters. Moreover, the entropy characteristics in the time domain are extracted along the time axis. The differences between short-circuit faults in the frequency domain are mainly reflected through the entropy value with limited expressive ability.

On the basis of feature extraction, the types of short-circuit faults can be recognized by classifiers. The methods used for constructing the classifier of short-circuit faults include neural networks (NN) [20], extreme learning machine (ELM) [21], support vector machine (SVM) [22], and so on. NNs have good robustness and adaptability resulting in it being widely used in the field of short-circuit fault classification [4,5]. However, it is difficult to determine the optimal structure of the NNs-based classifier and a large number of parameters need to be optimized. At the same time, the training for the NN processing requires a large number of historical samples which limit the applications of NNs. As the weights and thresholds are randomly generated in the process of network training, ELM has a faster learning speed. However, the diagnosis results are easily affected and fluctuated by both the hidden layer nodes and random parameters of ELM [23,24]. SVM has a good classification ability and robustness while the optimal factors of SVM can be easily chosen by the cross-validation method. In addition, it is easy to optimize the SVM with a small number of characters. The optimal classifier can be constructed by the cross-validation method to reduce the classification error due to the unreasonable parameters of SVM.

In order to improve the recognition accuracy of short-circuit fault signals, this paper presents a method for the detection and diagnosis of short-circuit faults based on EWT and LE. First, the fault signal is processed by the EWT method, with the results obtained by EWT being called IMFs. Subsequently, fault detection is realized by the modulus maxima point of the IMF_2_. Following this, the IMF_0_ of fault signals in the first period after the occurrence of the fault is decomposed into several time-frequency blocks of equal size. The local energy (LE) features are obtained by calculating the energy of each block to create the energy distribution of the fundamental frequency signals in the time domain. Finally, the SVM classifier is constructed according to the LE feature vectors to classify short-circuit faults. Comparison experiments verify the validity and creativeness of this new method.

## 2. Proposed Classification Framework for Short-Circuit Faults

**Figure 1:**
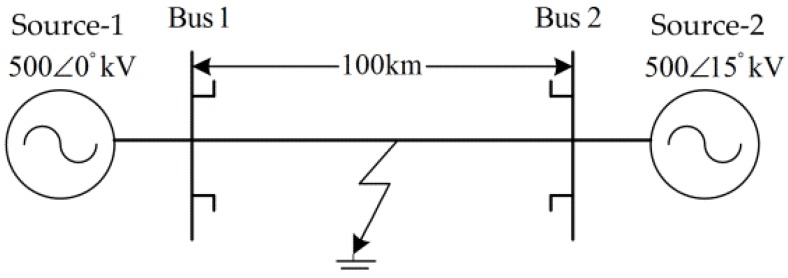
Diagram of the simplified transmission line model.

The real measured fault signals do not have the accurate fault time. In order to verify the accuracy of detection using the new approach and to obtain training samples for fault classification, the fault signals for analysis are first simulated with certain parameters. As shown in Figure 1, a 500 kV transmission system with double-terminal power supply and a system frequency of 50 Hz is adopted in this model, while PSCAD software is used to carry out the simulated experiments.

The transmission line parameters of the simulated system were obtained from a previous reference [5]. Positive, negative, and zero sequence parameters are shown in Table 1.

The ‘Bus 1’ is identified in Figure 1. Ten types of short-circuit faults are simulated, including single-phase grounding faults (AG, BG, and CG), two-phase grounding fault (ABG, BCG, and CAG), interphase short-circuit fault (AB, BC, and CA), and three-phase faults (ABC and ABCG) are simulated. The end of ‘Bus 1’ is found at the signal acquisition end and reference point for the fault distance.

The parameters of the short-circuit fault signals generated by the simulated system are set as follows:

1. The inception angle of voltage signals is set to a random integer value in the range of 0–360°;
2. The fault transition resistance is arranged as a random integer value in the range of 0–200 Ω;
3. The fault distance is set to a random integer value in the range of 10–90 km.

The ranges of the transition resistance and fault distance were both chosen according to the same paper [5]. The function of randi in MATLAB is used to generate the random value of three columns of random integers (100 integers for each column). Then the corresponding ranges for each column are 0–360, 0–200, and 10–90 and 100 kinds of fault condition are obtained for each fault type.

**Figure 2:**
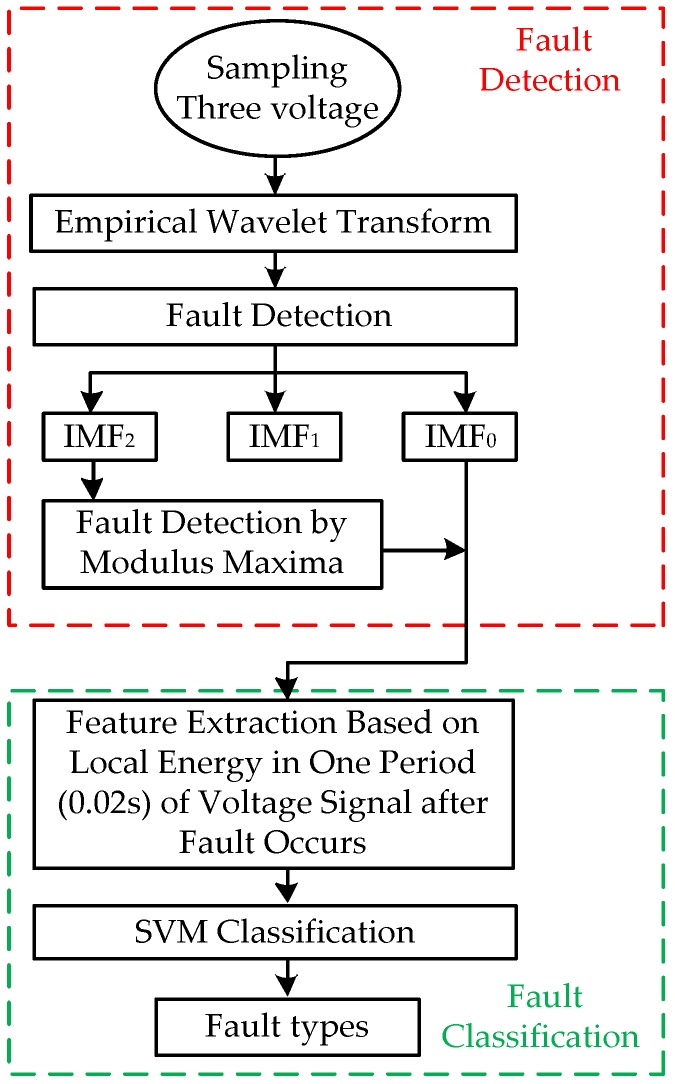
Flow chart of the proposed approach.

The process of detecting and diagnosing short-circuit faults proposed in this paper is shown in Figure 2. This mainly has two parts: the fault detection module and fault type identification module.

In the detection module, the short-circuit fault signals are decomposed by the EWT method. Following this, the modulus maxima value is used to determine the time that the fault occurred.

In the recognition module, the method calculates LE from IMF_0_ in the period after the occurrence of the fault. After this, the feature vector is used as an input for the classifier based on SVM to obtain the recognition result.

## 3. Processing of Short-Circuit Fault Signals by EWT and Detection of Faults

### 3.1. EWT

It is difficult to use the traditional EMD method to prove the orthogonality of the intrinsic mode function (IMF) components. There are some problems of mode mixing and pseudo-mode in the decomposition results. On the basis of adaptive orthogonal wavelet filter banks, the EWT method calculates the approximate and detail coefficients of the signals. Therefore, EWT obtains more accurate IMF components than EMD, making it more suitable for the analysis of short-circuit fault signals.

The number of IMFs from EWT can be determined in a specified or adaptive way. In this paper, an adaptive frequency domain segmentation method is employed with the specified number of IMFs. Since the default initial boundary of the divided spectrum used contains the default parameters of two values, three IMFs are obtained.

In this paper, the adaptive method is used to segment the original short-circuit fault signals f(n) in the frequency domain. Three IMFs ($f_{i}(n),1\leq i\leq 3$) were constructed to analyze the components of the short-circuit fault signals in different frequency domains.

(1) $$f(n)=\sum_{i=1}^{3}f_{i}(n)$$

The single-phase voltage signal f(n) (A-phase of CA fault) of short-circuit faults was used as an example to show the process of EWT analysis. The signal sampling frequency of short-circuit fault signals is 100 kHz. The sample of voltage signal contains 4000 sample points.

**Figure 3:**
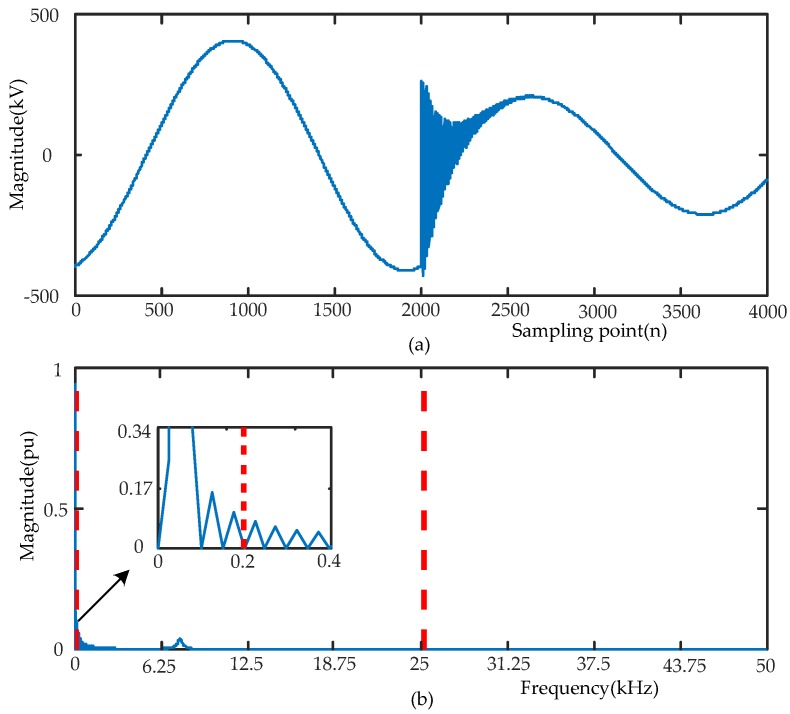
The signal of f(n) and Fourier spectrum with detected boundaries are listed as follows: (**a**) voltage signal; (**b**) detected Fourier supports for signal.

Firstly, the voltage signal is transformed by FFT to obtain the spectrum ([0, 50] kHz). Through an adaptive segmentation step of EWT, the segmentation boundary is obtained as follows: Ω_1_ = 0.2 kHz, and Ω_2_ = 25.275 kHz ($\Omega_{0}=0$ kHz, and $\Omega_{3}=50$ kHz). Since the frequency domain interval corresponding to each IMF can be expressed as $\Lambda_{i}=\;[\Omega_{i-1},\Omega_{i}]$, the segmentation intervals are determined by the segmentation boundary as $\Lambda_{1}=$ [0, 0.2] kHz, $\Lambda_{2}=\;$[0.2, 25.275] kHz and $\Lambda_{3}=$ [25.275, 50] kHz. The original signal and spectrum segmentation results are presented in Figure 3.

Secondly, a low-pass filter and two band-pass filters are defined based on the above segmentation boundary. The Fourier transformation expressions of the scaling function ${\hat{\phi }}_{1}\left(\omega \right),i=1$ and the empirical wavelet function ${\hat{\psi }}_{i}\left(\omega \right),i=2,3$ are respectively given as

(2) $${\hat{\phi }}_{1}\left(\omega \right)=\{\;\begin{array}{l} 1,\;\;\left|\omega \right|\leq \left(1-\gamma \right)\Omega_{1}\\ \cos\left[\frac{\pi }{2}\beta \left(\frac{1}{2\gamma \;\Omega_{0}}\left(\left|\omega \right|-\left(1-\gamma \right)\Omega_{1}\right)\right)\right],\left(1-\gamma \right)\Omega_{1}\leq \left|\omega \right|\leq \left(1+\gamma \right)\Omega_{1}\\ 0,\;\mathrm{otherwise} \end{array}$$

(3) $${\hat{\psi }}_{i}\left(\omega \right)=\{\begin{array}{c} \begin{array}{c} \begin{array}{c} 1,\;\left(1+\gamma \right)\Omega_{i}\leq \left|\omega \right|\leq \left(1-\gamma \right)\Omega_{i+1}\\ \cos\left[\frac{\pi }{2}\beta \left(\frac{1}{2\gamma \Omega_{i+1}}\left(\left|\omega \right|-\left(1-\gamma \right)\Omega_{i+1}\right)\right)\right],\left(1-\gamma \right)\Omega_{i+1}\leq \left|\omega \right|\leq \left(1+\gamma \right)\Omega_{i+1} \end{array} \end{array}\\ \begin{array}{c} \sin\left[\frac{\pi }{2}\beta \left(\frac{1}{2\gamma \;\Omega_{i}}\left(\left|\omega \right|-\left(1-\gamma \right)\Omega_{i}\right)\right)\right],\;\left(1-\gamma \right)\Omega_{i}\leq \left|\omega \right|\leq \left(1+\gamma \right)\Omega_{i}\\ \;0,\;\mathrm{otherwise} \end{array} \end{array}$$

where γ is a parameter that ensures no overlap between adjacent intervals [15]; and β(x) is an arbitrary function, which is defined as

(4) $$\beta (x)=\left\{ \begin{array}{ll} 0, & x\leq 0\\ & \beta (x)+\beta (x+1)=1,\;x\in [0,1]\\ 1, & x\geq 1 \end{array}\right.$$

Following this, an approximate coefficient can be obtained by computing the inner products of the empirical scaling function ϕ and signals f as shown in Equation (5). The detailed coefficients can be calculated according to Equation (6).

(5) $$W_{f}^{e}\left(1,n\right)=\langle f,\phi_{1}\rangle =\int f\left(\tau \right)\bar{\phi_{1}\left(\tau -n\right)}d\tau \;={\left(\hat{f}\left(\omega \right)\bar{{\hat{\phi }}_{1}\left(\omega \right)}\right)}^{\vee }\;i=1$$

(6) $$W_{f}^{e}\left(i,n\right)=\langle f,\psi_{i}\rangle =\int f\left(\tau \right)\bar{\psi_{i}\left(\tau -n\right)}d\tau ={\left(\hat{f}\left(\omega \right)\bar{{\hat{\psi }}_{i}\left(\omega \right)}\right)}^{\vee }\;i=2,3$$

where $\hat{g}$, $g^{\vee }$ and $\bar{g}$ denote the fast Fourier transformation, its inverse transformation, and complex conjugate of the function g respectively.

Finally, the IMFs of EWT are obtained as

(7) $$f_{1}\left(n\right)=W_{f}^{e}\left(1,n\right)*\phi_{1}\left(n\right)\;i=1$$

(8) $$f_{i}\left(n\right)=W_{f}^{e}\left(i,n\right)*\psi_{i}\left(n\right)\;i=2,3$$

where, ∗ is the symbol of convolution.

This shows that EWT can accurately extract the intrinsic mode information of different frequency components short-circuit fault signals. Since orthogonal filter banks are generated according to the spectral information of the original fault signals, this approach is more adapted for processing short-circuit fault signals without the influence of pseudo-modes.

### 3.2. Processing of Short-Circuit Fault Signals Processing Based on EWT

**Figure 4:**
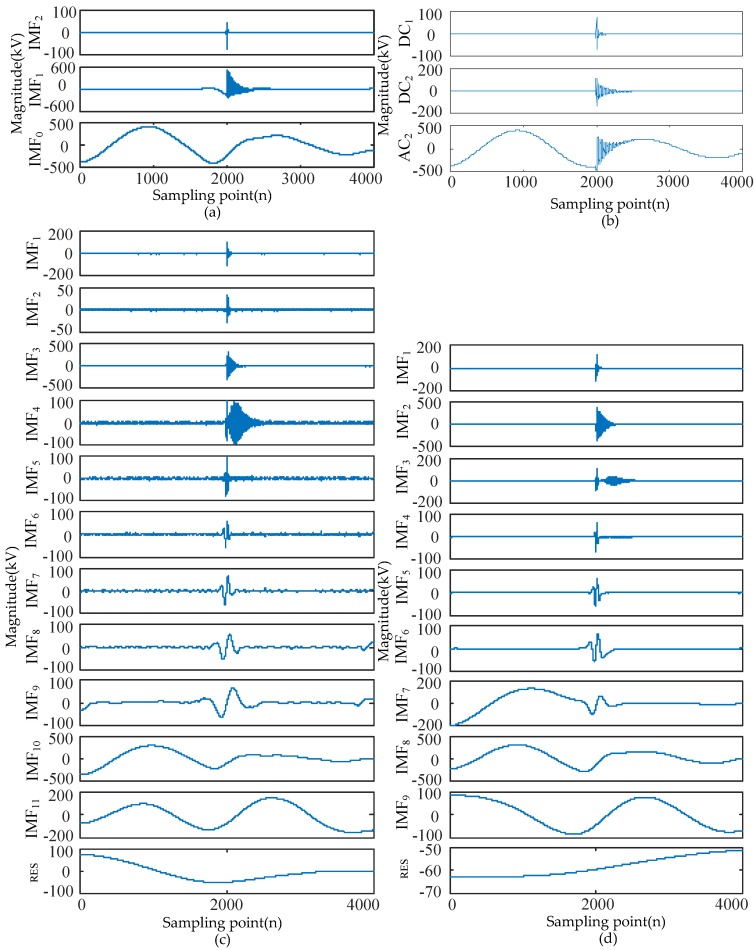
Comparison experiments of different signal processing methods for fault signal analysis are shown as follows: (**a**) modes extracted by EWT; (**b**) modes extracted by WT; (**c**) modes extracted by CEEMDAN; (**d**) modes extracted by Improved CEEMDAN.

In order to verify the advancement of EWT, four methods including EWT, WT, CEEMDAN, and improved CEEMDAN are used to process the short-circuit fault signals. The parameters of the four methods are set as follows. EWT is set in Section 3.1, while the number of decomposed layers of WT is set to two. Thus, this results in three components for achieving a comparison with EWT using the same number of components. CEEMDAN and improved CEEMDAN adopt default parameter. By comparing the effect and computing time of different signal processing methods, the effectiveness and advancement of EWT are verified. Figure 4 shows the decomposition results of short-circuit fault signals by different methods.

As shown in Figure 4, modes extracted by EWT, WT, CEEMDAN, and improved CEEMDAN approaches are described respectively. EWT decomposes the original fault signal into three IMFs as shown in Figure 4a. Approximate coefficient (AC_2_) and detailed coefficients (DC_1_ and DC_2_) are obtained by WT. However, more IMFs are extracted by CEEMDAN and the modes contain residual noise. These defects cause difficulties in feature extraction. The improved CEEMDAN method effectively suppresses the residual noise in modes and improves the performance of CEEMDAN method, although it still results in a larger number of IMFs compared to EWT.

The time required for the four methods to decompose the same fault signal is 0.042 s, 0.038 s, 654.662 s, and 518.956 s. Compared to CEEMDAN and improved CEEMDAN method, EWT has a higher computational efficiency. Moreover, the computation efficiency of EWT is slightly less than WT.

IMF_0_ are used to extract features in Section 4.1 and thus we compared these components. As seen in Figure 4, the IMF_0_ extracted by EWT does not contain other components that form the foundation for feature extraction. The frequency domain is segmented in a restricted manner after the number of decomposition levels in WT is fixed. However, the IMF_0_ AS_2_ contains an oscillating component which is not conducive for feature extraction. In comparison, the IMF_10_ extracted by CEEMDAN and the IMF_8_ extracted by improved CEEMDAN does not contain other components, although both methods require more time to process the fault signal. Therefore, EWT was chosen as the signal processing method.

### 3.3. Detection of Short-Circuit Faults Based on EWT

After obtaining the results of short-circuit fault signals processed by EWT, the time-frequency matrix composed by IMFs can be used for feature extraction. If the features are extracted from the whole time-frequency matrix, there will be no obvious fault characteristics with a high dimension of feature vectors. Moreover, it will increase the complexity of the classifier and reduce the classification accuracy. In the present research, the range of feature extraction is reduced to one period with the most transient information after the occurrence of the fault [25]. Thus, the feature dimension of short-circuit fault signals can be reduced effectively.

In order to obtain the singularity of fault signal simply and clearly, the modulus maxima (MM) of wavelet transform is introduced. The MM is only valid if it meets the following condition [26]:

∀ε>0, a neighborhood $\left|t-t_{o}\right|<\varepsilon$ exists; for every $t-t_{o}$.

(9) $$\left|f(j,t_{0})\right|\geq \left|f(j,t)\right|$$

The time that a fault occurs often corresponds to the singular point of the voltage signal. The traditional WT has good spatial localization properties, which can accurately detect the singularity of fault signals. Therefore, the time that a fault occurs can be pinpointed by the modulus maxima point in high frequency mode components [27]. A previous study [15] pointed out that EWT is based on the wavelet theory framework. Furthermore, the largest difference between EWT and WT is that EWT is based on the original signal for constructing orthogonal wavelet which does not require the mother wavelet to be chosen. Thus, the fault time can be located through the modulus maxima point in the high-frequency mode component based on EWT. This lays the foundation for extracting features of short-circuit fault signals based on the first cycle after the occurrence of the failure.

**Figure 5:**
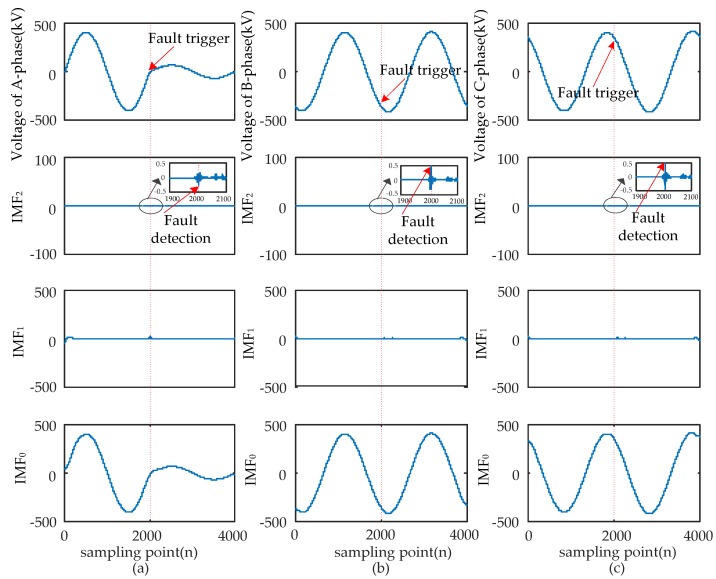
AG fault signal’s modes extracted by the EWT are shown as follows: (**a**) A-phase voltage and its IMFs; (**b**) B-phase voltage and its IMFs; (**c**) C-phase voltage and its IMFs.

**Figure 6:**
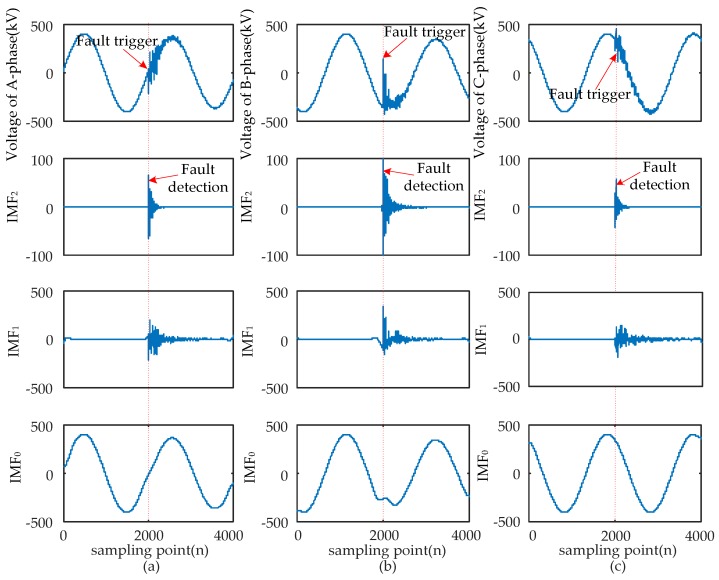
ABG fault signal’s modes extracted by the EWT are shown as follows: (**a**) A-phase voltage and its IMFs; (**b**) B-phase voltage and its IMFs; (**c**) C-phase voltage and its IMFs.

**Figure 7:**
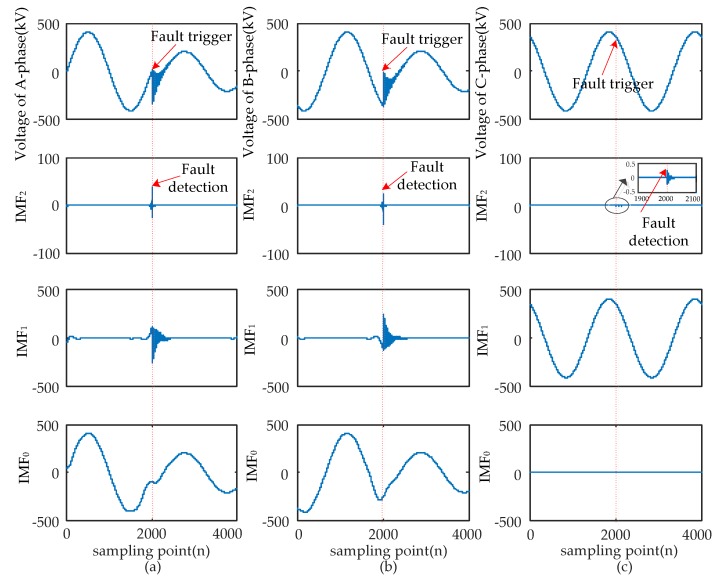
AB fault signal’s modes extracted by the EWT are shown as follows: (**a**) A-phase voltage and its IMFs; (**b**) B-phase voltage and its IMFs; (**c**) C-phase voltage and its IMFs.

**Figure 8:**
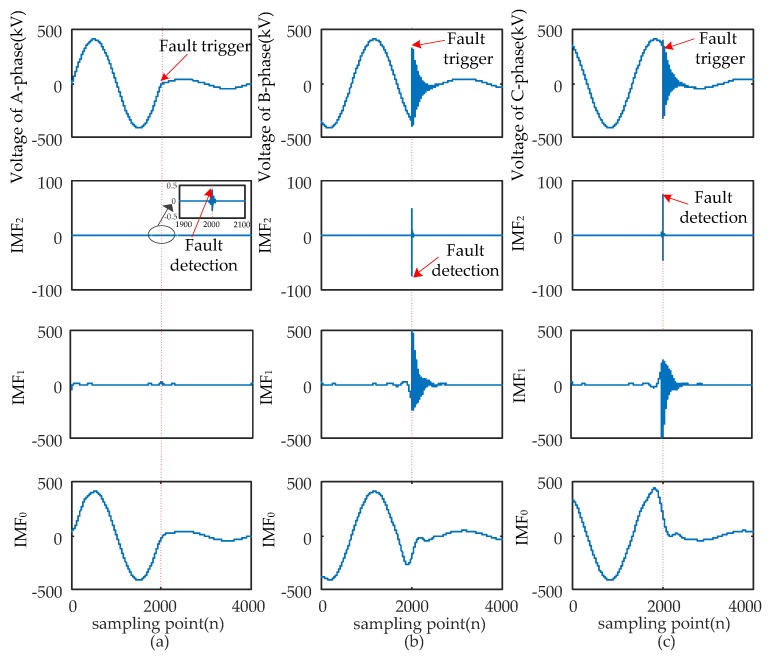
ABC fault signal’s modes extracted by the EWT are shown as follows: (**a**) A-phase voltage and its IMFs; (**b**) B-phase voltage and its IMFs; (**c**) C-phase voltage and its IMFs.

When short-circuit faults occur, the voltage signals of the fault and non-fault phases change together. Therefore, the time that a fault occurred should be determined synthetically by the modulus maxima of IMF_2_ from three-phase signals. Four typical types of short-circuits include the single-phase grounding fault (AG), two-phase grounding fault (ABG), interphase short-circuit fault (AB), and three-phase fault (ABC and ABCG). These were used to verify the ability of EWT to pinpoint time and location. The transform results are shown in Figure 5, Figure 6, Figure 7 and Figure 8 and uniform scale is adopted.

As shown in Figure 5, Figure 6, Figure 7 and Figure 8, the phase voltage signal of four types of faults are decomposed into three IMFs (IMF_2_, IMF_1_, and IMF_0_) by EWT. Compared with IMF_0_ and IMF_1_, the modulus maxima of IMF_2_ is more obvious. Therefore, the fault time can be determined by the modulus maxima point of IMF_2_. At the same time, there are modulus maxima in the three-phase signal when short-circuit faults occur. Therefore, the occurrence time of short-circuit faults can be determined by the modulus maxima of IMF_2_ from the three-phase voltage signals processed by EWT.

The detailed process of the new fault detection method based on the modulus maxima of IMF_2_ decomposed by EWT is described as follows:

1. If the result of fault detection from different phase signals is consistent, this result is considered the fault detection result (FDR);
2. If two values of the three detection results are the same, the same value is decided as the FDR;
3. If the three-phase detection results are different, the minimum detection value is taken as the FDR.

The detection results of the three methods for different types of short-circuit faults are shown in Table 2.

In order to verify the effectiveness and advancement of the new method, the location results of the new method are compared with other methods based on Shannon energy entropy (EE) [5] and Shannon singular entropy (SE) [17].

As shown in Table 2, the error of fault detection with the ‘EWT + MM’ approach for the AG type is 2 sampling points (20 us), while the error of ABG, AB, and ABC is 0. The detection error of the ‘EWT + SE’ and ‘EWT + EE’ method is larger than that of the ‘EWT + MM’ method.

## 4. Feature Extraction Based on Local Energy

### 4.1. Local Energy

Existing research has shown that short-circuit fault signals in the first period change dramatically when a fault occurs [25,27]. Moreover, the signal in this period contains the most abundant fault features. In this paper, it is found that the IMF_0_ based on EWT can reflect the changing trends of fault signals.

After obtaining the time at which a short-circuit fault occurs, the first period of the time-frequency matrix after the occurrence of a fault is used to extract the features for constructing the feature vector of the classifier. In order to present the change in the IMF_0_ over time, the new method uses local energy (LE) to construct feature vectors. The time-frequency vector obtained by EWT is divided into time-frequency blocks of equal size. Following this, the LE is obtained from each time-frequency block. Finally, the feature vector of short-circuit fault signals is composed by combining the LE of all time-frequency blocks.

**Figure 9:**
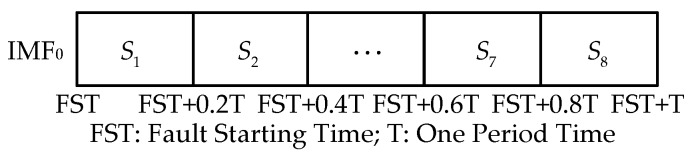
The segmentation of time-frequency plane.

In this paper, the sampling rate of short-circuit fault signals is 100 kHz. In the feature extraction step of the new approach, the IMF_0_ obtained by EWT constitutes the vector E. The vector E is the time-frequency vector having a dimension of 1×2000 is divided into eight equal-sized time-frequency blocks along the time axis, so that every block has 400 sampling points (0.2 T) as shown in Figure 9.

The energy of the time-frequency block $S_{1},S_{2},\cdots ,S_{8}$ is described as $Z_{1},Z_{2},\cdots ,Z_{8}$ respectively. These values are calculated as

(10) $$Z_{u}=\sum {\left|E_{v}\right|}^{2}\;u=1,\cdots ,8;v=1,\cdots ,125$$

where $E_{v}$ is the sampling point of vector E.

The features of three-phase voltage signals are calculated according to Equation (9) to obtain the feature vector $Z=[Z_{A}\;Z_{B}\;Z_{C}]=[Z_{1},\cdots ,Z_{24}]$. The feature vector reflects the energy variation characteristics of the IMF_0_ of three-phase signals from short-circuit faults in the time domain.

### 4.2. Feature Extraction Based on LE

**Figure 10:**
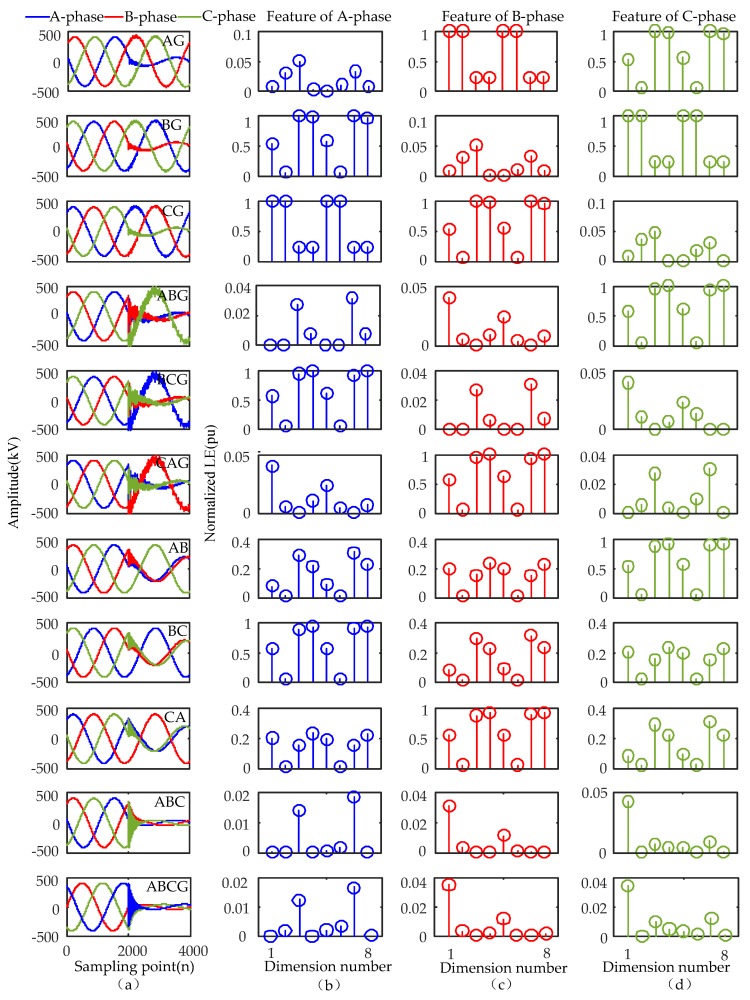
Three-phase voltage and its LE features of different short-circuit faults are listed as: (**a**) three-phase voltage signals of 10 types of faults; (**b**) LE features corresponding to A-phase voltage signal; (**c**) LE features corresponding to B-phase voltage signal; (**d**) LE features corresponding to C-phase voltage signal.

Ten types of short-circuit fault signals in Figure 10a are listed to illustrate the feature extraction process. Their parameters are set as follows: fault distance is 30 km, the fault initial angle is 0°, and the transition resistance is 100 Ω. The results of feature extraction are shown in Figure 10b–d.

The black, red, and green discrete points correspond to the LE features of the IMF_0_ of A, B, and C-phase voltage signals, respectively.

As shown in Figure 10, the feature amplitude of the IMF_0_ of the non-fault phases (B and C) is significantly larger than that of the fault phase A once an AG fault occurs. These characteristics meet the trend characteristics that the fault phase voltage amplitude decreases and non-fault phase amplitude increases when a single-phase ground fault occurs. The feature amplitude relationship between the non-fault phase and fault phase has the same conclusion as AG when BG and CG faults occur.

When the ABG fault happens, the feature values of the fault phases (A and B) are small, while the feature values of the non-fault phase C are large. These characteristics meet the trend characteristics that the fault phase voltage amplitude decreases and the non-fault phase amplitude increases when a two-phase ground fault occurs. The feature amplitude relationship between the non-fault phase and fault phase have the same conclusion as ABG when the BCG and CAG fault occur.

When the AB fault happens, the feature values of the fault phases (A and B) are small, while the characteristic values of the fault phase C are large. These characteristics meet the trend characteristics that the fault phase voltage amplitude decreases and the non-fault phase amplitude increases when an interphase short-circuit fault occurs. The feature amplitude relationship between the non-fault phase and fault phase has the same conclusion as AB when the BC and CA faults occur.

When the ABC fault happens, the feature values of the three phases are small, which is consistent with previous findings that the amplitude of the three-phase voltages decrease. The feature values and trend characteristics of three-phase voltage signals of ABC and ABCG are relatively close under the same fault condition.

The values of the input classifier are arranged according to the feature values of the A, B, and C phases.

As shown in the above analysis, the energy distribution characteristics of different types of short-circuit fault can be clearly reflected by the LE energy features in three-phase signals. There are obvious differences in the feature values of 10 types of faults. All the above characteristics provide a significant basis for the identification of fault type. The classification ability with LE features in the new method will be further validated through statistical experiments in Section 6.

In order to verify the effectiveness of the feature extraction method, the EE [5] and SE [17] feature extraction methods were also employed for comparison with the proposed method. Experiments show that every short-circuit fault signal obtains three IMF components based on EWT. The time-frequency characterization of signals using EE and SE based on the whole time-frequency domain is limited. In comparison, the LE feature characteristics reflect the energy change in the IMF_0_ of the signal in the time domain, while the time domain characteristic of short-circuit fault signals is more detailed. A detailed simulation is shown in Section 6.3.

## 5. Design of Short-Circuit Fault Classifier Based on SVM

**Figure 11:**
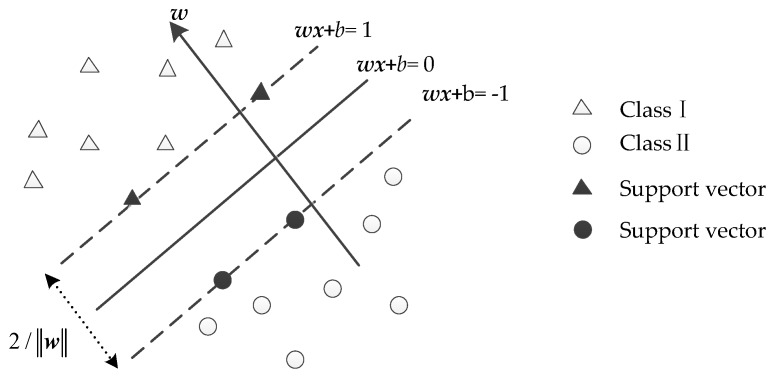
Classification principle of SVM.

In the existing algorithms used for short-circuit fault classifiers, SVM performs accurately in classification and has robustness with less training samples. The goal of SVM is to classify the data points belonging to different classes by constructing the optimal hyperplane [28]. Figure 11 presents the classification principles of SVM.

In order to better understand the basic principles of SVM, the two-class linearly separable data set $E^{\prime }$ is assumed as

(11) $$E^{\prime }={\{x_{k},y_{k}|x_{k}\in R^{U},y_{k}\in \{-1,1\}\}}_{k=1}^{N}$$

where $x_{k}$ is a *U*-dimensional input vector; $y_{k}$ is the corresponding class of $x_{k}$; and *N* is the number of samples. The data points in set *E* can be divided into two types by the hyperplane, which is defined as

(12) wx+b=0

where ***w*** is the weight vector; and *b* is the scalar. Two types of sample vectors from the hyperplane are called support vectors. The distance between the two-class support vectors and separating hyperplane is called the separating margin which is given as

(13) $$m=\frac{2}{\left\|w\right\|}$$

In order to get the maximized *m*, we need to minimize the ‖w‖. The goal of SVM is to determine the value of ***w*** and *b*, so that the separating margin is the largest. The optimal separating margin can be obtained by solving the quadratic optimization problem as

(14) $$\left\{ \begin{array}{l} \min\;\frac{1}{2}{\left\|w\right\|}^{2}\\ \mathrm{subject}\;\mathrm{to}\;y_{k}(wx_{k}+b)\geq 1,\;k=1,2,\cdots ,N\; \end{array}\right.$$

In order to solve the above problems, the Lagrange multiplier α is introduced, and the optimization objective function is obtained by

(15) $$\left\{ \begin{array}{l} \max\;L^{\prime }(\alpha )=\;\sum_{k=1}^{N}\alpha_{k}-\frac{1}{2}\sum_{k=1,g=1}^{N}\alpha_{k}\alpha_{g}y_{k}y_{g}x_{k}^{T}x_{g}\\ \mathrm{subject}\;\mathrm{to}\;\alpha_{k}y_{k}=0 \end{array}\right.$$

After solving the above optimization problems, we can obtain the optimal solution $\alpha_{k}^{*}$, before calculating the best solutions for $w^{*}$ and $b^{*}$. Finally, the optimal classification function is

(16) $$g^{\prime }(x)=\mathrm{sgn}((\sum_{k=1}^{N}\alpha_{k}^{*}y_{k}x_{k}^{T}x_{g}+b^{*}))$$

In fact, the classification problem of short-circuit faults is a non-linear separable problem. We can map the sample data from the low-dimensional space to a high-dimensional space by the kernel function K. By replacing $x_{k}^{T}x_{g}$ with $K(x_{k},x_{g})$, the optimal classification function is obtained as

(17) $$g(x)=\mathrm{sgn}((\sum_{k=1}^{N}\alpha_{k}^{*}y_{k}K(x_{k},x_{g})+b^{*}))$$

As the feature space corresponding to the Gauss kernel function is infinitely dimensional, the finite sample is linearly separable in the space. In this paper, Gauss kernel function is used as

(18) $$K(x_{k},x_{g})=\exp(-\gamma {\left\|x_{k}-x_{g}\right\|}^{2})$$

where γ is the parameter of the Gauss kernel function.

From the above analysis, we can know that SVM is limited in only being able to deal with the binary classification. SVM needs to be further improved in order to solve the multi-classification problem.

In this paper, the LIBSVM package based on “one-against-one” structure [29] is used to solve the problem of multi-classification problem of short-circuit faults.

## 6. Simulation and Analysis

A total of 1100 samples (100 samples for each fault type) are generated in the simulated system shown in Figure 1. Through statistical experiments, the number of training samples for the classifier of SVM is determined to be 660 (60 samples for each fault type). In particular, the training samples are randomly selected.

### 6.1. Comparison of Fault Detection Effect for Short-Circuit Fault Signals

In order to further verify the fault location efficacy of the new method, 1000 samples are tested. The statistical experiments validated the advancement of the new method. The test results are shown in Table 3.

Table 3 lists the fault detection results of 10 types of short-circuit fault signals with noise signals of 0, 38, and 57 dB when the ‘EWT + MM’, ‘EWT + SE’ and ‘EWT + EE’ methods are used to deal with the signals, respectively. The average error of the fault detection using the ‘EWT + MM’ method is minimal.

It can be seen from Table 3 that the detection error of ‘EWT + MM’ is lower than that of the ‘EWT + SE’, and ‘EWT + EE’ methods when 0, 38, and 57 dB noise are added to short-circuit fault signals. The mean detection error of short-circuit fault signals increases with the addition of noise. Furthermore, it was concluded that a greater noise intensity resulted in a greater overall average detection error.

### 6.2. Setting Parameters for Classifier

In order to avoid the influence of the unreasonable parameters for the classification accuracy of the SVM classifier, this paper uses the cross-validation method to optimize the parameters of *C* and γ in SVM. A five-fold cross-validation method is used to choose the optimal value of parameters *C* and γ under different dimensions of LE features, where $C\in \left\{2^{12},2^{11},\cdots ,2^{-1},2^{-2}\right\}$ and $\gamma \in \left\{2^{4},2^{3},\cdots ,2^{-9},2^{-10}\right\}$. The number of time-frequency blocks and feature dimensions are shown in Table 4. The classification accuracy of the 225 parameter combinations in SVM is tested with different LE feature vectors. The optimal parameters and the classification accuracy in different feature dimensions are shown in Table 4.

From Table 4, we can find the following characteristics:

1. When the number of blocks is increased from 1 to 8 in each phase, there is a dramatic increase in classification accuracy. In particular, the accuracy rate is highest (99.77%) when there are 8 blocks and the parameters of *C* and *γ* are 2^8^ and 2^−5^.
2. The accuracy rate is generally stable when the number of blocks changes from 8 to 2000.
3. When the number of blocks (feature dimension) is increased, there is greater complexity in the classifier. Therefore, the feature dimensions, *C* and *γ* in SVM are set as 24, 2^8^, and 2^−5^ in the new method.

### 6.3. Comparison of Short-Circuit Faults Classification Methods

Based on the above analysis, we can determine the advantages of the signal processing method of EWT and the fault detection method used in the new method. In order to further verify the effectiveness of the new method in classifying short-circuit faults, the features extracted by LE, SE, and EE with classifiers of SVM, BPNN (Back-Propagation Neural Network), and ELM are combined to create a different classification method for short-circuit signals. Following this, the classification accuracy of these methods is compared.

In order to ensure the credibility of this comparison, the parameters of the classifier are set as follows. When different features are used, the dimensions of the input vector and related parameters in SVM, ELM, and BPNN are determined in the same way as described in Section 6.2. In the ELM classifier, the number of input nodes represents the input feature dimension, while the number of output nodes is the number of fault classes. The activation function in ELM is the sigmoid function $f(x)=1/(1+e^{-\lambda x})$. Thus, two parameters need to be determined: the number of nodes $\tilde{N}$ in the hidden layer and the parameter *λ* that determines the smoothness of the sigmoid function. The optimal combination of $\tilde{N}$ and *λ* is obtained by the grid search method, where $\tilde{N}\in \left\{2,4,\cdots ,28,30\right\}$ and $\lambda \in \left\{10^{-1},10^{-2},\cdots ,10^{-9},10^{-10}\right\}$. The structure parameters of ELM and BPNN are shown in Table 5. The optimal training parameters of BPNN such as learning rate, learning rate reduced factor, learning rate minimum bound, and number of epochs are obtained by five-fold cross-validation [30]. The training parameters of BPNN are as follows: the maximum epoch number is 1000; the learning rule is Levenberg–Marquardt; the moment constant is 0.98; the mean squared error is 1.00 × 10^−5^ [31]. The classification results of different methods are shown in Table 6.

As shown in Table 6, the new method using LE has higher classification accuracy compared to the results from ‘EWT + LE + SVM’, ‘EWT + SE + SVM’ and ‘EWT + EE + SVM’. In addition, this same conclusion was obtained after comparing the classification results of ‘EWT + LE + ELM’, ‘EWT + SE + ELM’ and ‘EWT + EE + ELM’ or comparing the recognition results of ‘EWT + LE + BPNN’, ‘EWT + SE + BPNN’ and ‘EWT + EE + BPNN’. Therefore, the methods with LE features have the greatest accuracy for classification. These results verify the effectiveness and advancement of the LE features for the classification of short-circuit faults.

At the same time, the experimental results in Table 6 show that the proposed method using ‘EWT + LE + SVM’ has the highest recognition accuracy. The overall accuracy rate is 99.77% and the accuracy rate for each fault is 100% except ABC/ABCG fault (98.75%) which verifies that the new method using SVM to construct short-circuit faults classifiers is more feasible.

In addition, based on the same set of LE features, the calculation time of the three classifiers is shown in Table 7. The experimental platform is a PC with i3 processor (2.3 GHz frequency) and 4 G memory.

From Table 7, the training and testing time of SVM are both is obviously shorter than BPNN and longer than that of ELM. Considering the efficiency and accuracy of classification, it is more reasonable to use SVM for realizing the classification of short-circuit faults, with these results verifying the effectiveness and progressiveness of the new method.

In order to validate the noise immunity of the proposed classification method ‘EWT + LE + SVM’, different noise level are added to the original short-circuit fault signals. The experimental results are shown in Table 8.

As shown in Table 8, the classification results still have a high recognition accuracy (higher than 98.86%) in environments with different noises at 27, 33, 38, 43, and 57 dB. Thus the noise immunity of the proposed classification method has been further verified. The fault type of ABC and ABCG are recognized as one class because of the similar feature values and trend characteristics [1].

The effectiveness and advancement of the new method are verified by the experimental results. In order to further verify the effectiveness of the proposed method, the practice short-circuit fault signals are tested.

## 7. Verification of Actual Signals

In order to verify the recognition capability of the new approach for actual signals, the short-circuit fault signals collected in line substation were used for tests.

**Figure 12:**
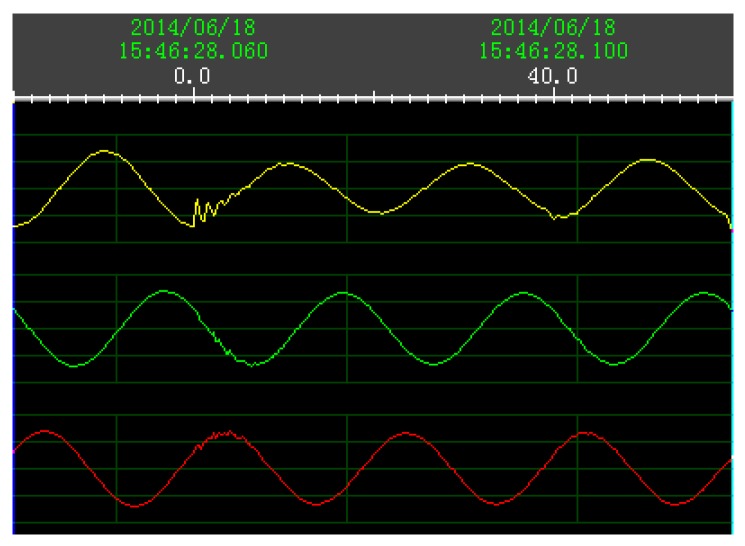
Waveform of three-phase voltages when AG fault occurred.

From 7 March 2012 to 9 August 2014, a total of 167 sets of transmission line fault signals were collected from the photoelectric voltage transformer with a serial number of OET711AVTZ in four 220 kV substations. In the serial number of the photoelectric voltage transformer, “OET” is the short name of the photoelectric transformer; “7” is the design serial number; “11” is the abbreviation of 110 kV; “A” means that this will be used as a communication system; “VT” is the voltage transformer type; and “Z” means that it is a hanging device. The fault voltage signals are recorded in the digital fault recorder system. A set of phase voltage signals with faults are shown in Figure 12.

The details of the real measured data set obtained by the photoelectric voltage transformer are as follows:

- Fault lines: 110 kV transmission lines;
- Fault type: a total of 10 types of short-circuit fault;
- Collection site: 110 kV bus side in 220 kV substation;
- Signal sampling rate: 5000 Hz.

The training set is constructed by using the descending sampling rate simulation signal, while the training model is constructed by using the recognition scheme proposed in Section 6.3.

### 7.1. Detection of Actual Short-Circuit Faults

**Figure 13:**
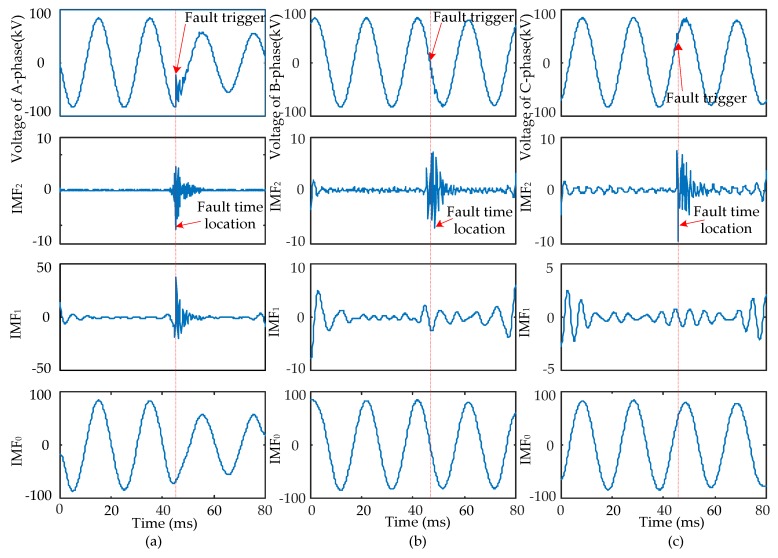
Actual AG fault signal’s modes extracted by the EWT are shown as follows: (**a**) A-phase voltage and its IMFs; (**b**) B-phase voltage and its IMFs; (**c**) C-phase voltage and its IMFs.

**Figure 14:**
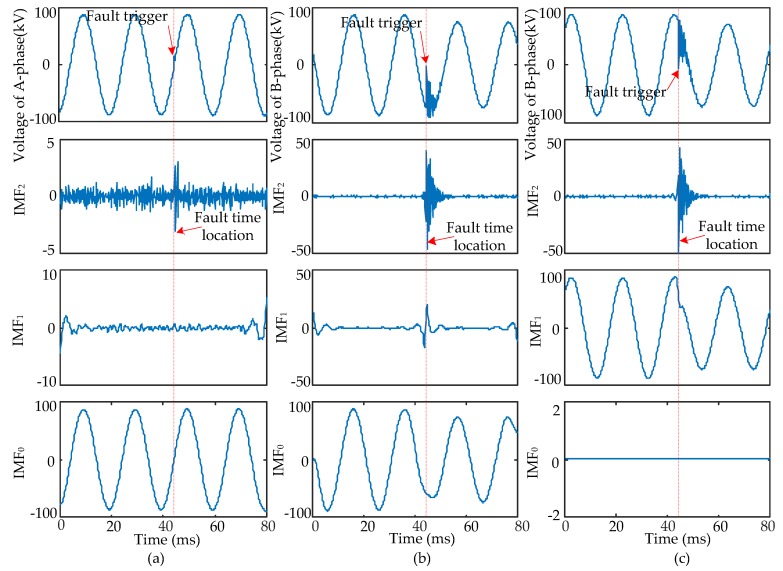
Actual BCG fault signal’s modes extracted by the EWT are shown as follows: (**a**) A-phase voltage and its IMFs; (**b**) B-phase voltage and its IMFs; (**c**) C-phase voltage and its IMFs.

**Figure 15:**
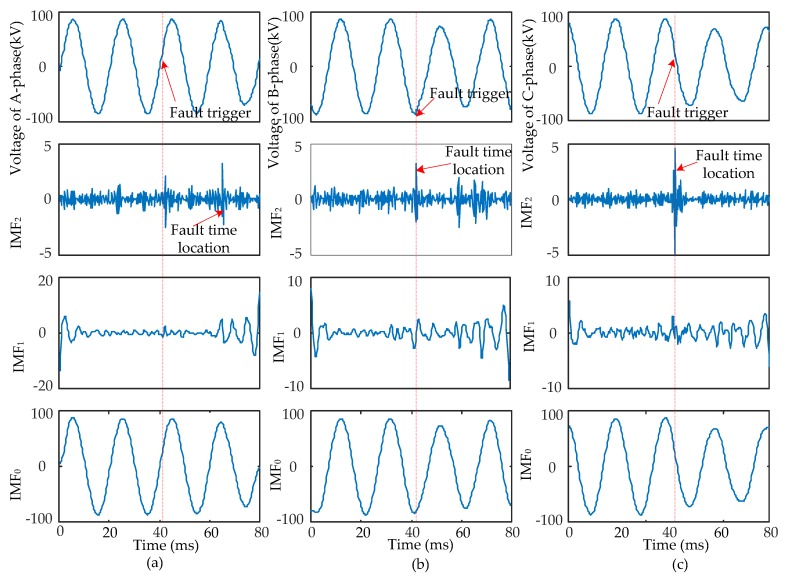
Actual BC fault signal’s modes extracted by the EWT are shown as follows: (**a**) A-phase voltage and its IMFs; (**b**) B-phase voltage and its IMFs; (**c**) C-phase voltage and its IMFs.

**Figure 16:**
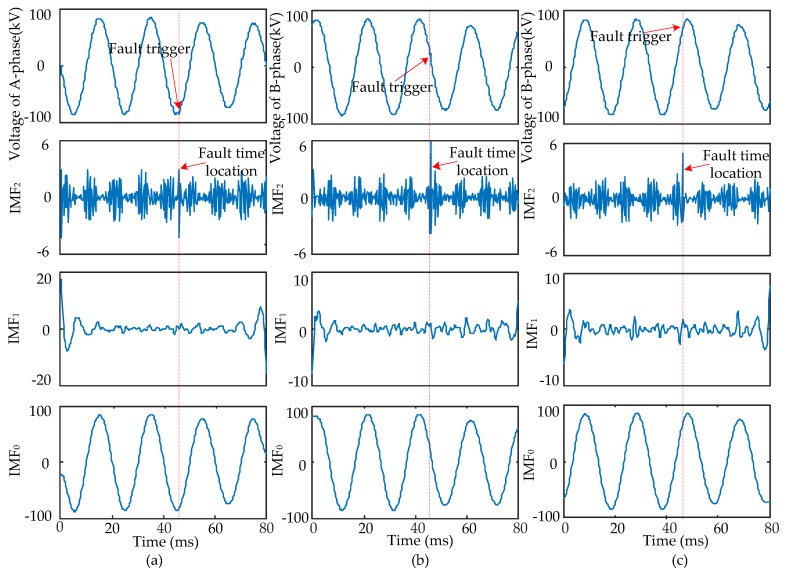
Actual ABC fault signal’s modes extracted by the EWT are shown as follows: (**a**) A-phase voltage and its IMFs; (**b**) B-phase voltage and its IMFs; (**c**) C-phase voltage and its IMFs.

The EWT is tested by using four types of fault types AG, BCG, BC, and ABC as examples. The results are shown in Figure 13, Figure 14, Figure 15 and Figure 16.

We further verified the accuracy of the ‘EWT + MM’ method in pinpointing the time that a short-circuit fault occurred. As shown in Figure 13, Figure 14, Figure 15 and Figure 16, each signal of 4 fault types are decomposed into three IMFs (IMF_2_, IMF_1_, and IMF_0_) by EWT. In particular, the IMF_1_ in Figure 14c is different from that in Figure 14a,b. The reason is that when the C-phase voltage signal is processed by EWT, this results in a narrow interval for the frequency domain corresponding to IMF_0_ while the components at a frequency of 50 Hz appears in the interval of IMF_1_. The theory of ‘EWT + MM’ in Section 3.3 was used for fault detection.

The test results of the ‘EWT + MM’ method being used to detect short-circuit faults are shown in Table 9.

As shown in Table 9, the detection results of three-phase voltage signals of the AG faults are different. The minimum value of 45.2 in the A-phase represents the FDR, which is close to the real value (Figure 13). When the BCG fault occurs, the FDR of the C-phase in the BCG fault is the smallest detection result of 44.4, which is close to the true value in Figure 14. As shown in Figure 15, there is big difference between the detection result of A-phase and the real failure time. While the detection value of B-phase and C-phase of the BC fault are the same (42.8), FDR has a value of 42.8. As shown in Figure 16, the FDRs of the B-phase and C-phase have the same value of 42.8, which represents the FDRs coming close to their true value.

### 7.2. Classification of Short-Circuit Faults

After obtaining the time that a short-circuit fault occurred, the first period of the IMF_0_ after the occurrence of this fault is used to extract features. Following this, the feature vector is used as an input for the trained model in Section 6 to realize fault classification.

The following aspects require special instructions:

1. The IMF_0_ is needed, with the IMF_1_ of C-phase voltage signal in Figure 14c representing the IMF_0_. Since statistical experiments show that the amplitude of IMF_0_ is less than 2 in this case, the threshold of 10 is set for the purpose of uniform processing and easy calculation. If the amplitude of the IMF_0_ is less than 10, the IMF_1_ is treated as the IMF_0_.
2. The IMF_0_ of the real signals were normalized.
3. The simulation samples are reduced to 5000 Hz and IMF_0_ were normalized.

The experimental results are shown in Table 10. The advancement of the fault classification method proposed in this paper is verified.

As shown in Table 10, the classification method proposed in this paper has high accuracy in identifying faults for real signals. Therefore, the advanced nature of the proposed method is validated.

## 8. Conclusions

In this paper, a new classification method of short-circuit faults in the electric transmission line based on EWT and LE is proposed. The new method has the following advantages:

1. Compared to the CEEMDAN and the improved CEEMDAN method for the decomposition of short-circuit fault signals, EWT has a smaller number of IMFs and the decomposition result has a higher accuracy. Therefore, it is more suitable for the processing of short-circuit fault signals;
2. The new method directly uses the MM of IMF_2_ in three-phase voltage signals to determine the failure time. There is no need to use other complex methods for fault detection and this creates the foundation for feature extraction;
3. Compared to entropy features, the feature of LE reduces the complexity of computation. The change in IMF_0_ in short-circuit fault signals can be presented in the time domain which is conducive to the accurate identification of short-circuit fault signals. More importantly, the technique enhances the noise immunity of the fault classification scheme.

In the future work, the proposed method can be compiled into software and applied for real-time data analysis.

## Figures and Tables

**Table 1:** Parameters of transmission line.

Parameters	Value
Positive and negative sequence resistance (Ω/km)	0.035
Positive and negative sequence inductance (Ω/km)	0.424
Positive and negative sequence reactance (S/km)	2.726 × 10^−6^
Zero sequence resistance (Ω/km)	0.3
Zero sequence inductance (Ω/km)	1.143
Zero sequence reactance (S/km)	1.936 × 10^−6^

**Table 2:** Comparison of short-circuit fault detection results with different methods.

Method	Type Fault	Number of Sampling Point	Fault Detection Time of Each Phase (Sampling Point)			FDR (Sampling Point)	Error (Sampling Point)
			A-Phase	B-Phase	C-Phase		
EWT + MM	AG	2001	2006	2003	2003	2003	2
	ABG	2001	2001	2002	2009	2001	0
	AB	2001	2001	2000	2001	2001	0
	ABC	2001	2001	2000	2001	2001	0
EWT + SE	AG	2001	2011	2013	2011	2011	10
	ABG	2001	2009	2014	2008	2008	7
	AB	2001	2005	2007	2006	2005	4
	ABC	2001	2012	2007	2007	2007	6
EWT + EE	AG	2001	2016	2021	2017	2016	15
	ABG	2001	2018	2016	2015	2015	14
	AB	2001	2014	2012	2012	2012	11
	ABC	2001	2018	2018	2020	2018	17

**Table 3:** The mean fault detection error of different detection methods under different noise.

Fault Type	Mean Error (ms)								
	EWT + MM			EWT + SE			EWT + EE		
	0 dB	38 dB	57 dB	0 dB	38 dB	57 dB	0 dB	38 dB	57 dB
AG	0.2697	0.3513	0.2925	0.4715	2.8234	0.5274	1.7121	4.1411	1.7857
BG	0.2782	0.3845	0.2738	0.5014	2.5281	0.5587	1.6547	4.4726	1.8586
CG	0.2710	0.3651	0.2715	0.4637	2.8965	0.5341	1.7983	4.2705	1.9515
ABG	0.3247	0.3613	0.3492	0.7452	3.2739	0.9102	1.8109	4.3162	2.2711
BCG	0.2816	0.3216	0.2979	0.7518	3.3518	0.8853	1.3152	3.5790	1.5196
CAG	0.2793	0.3304	0.3153	0.7013	2.9573	0.8302	1.3785	3.6527	1.3904
AB	0.2464	0.3679	0.3495	1.6241	3.8901	2.3217	2.1251	4.1793	2.1352
BC	0.2358	0.3913	0.3718	1.7285	4.1357	2.4901	1.8347	3.7201	2.0274
CA	0.2419	0.3817	0.3552	1.7528	3.6569	1.9563	1.8629	4.2705	2.2853
ABC	0.3356	0.3674	0.3576	0.5222	2.6948	0.5673	0.8706	2.6023	1.1070
ABCG	0.3401	0.3627	0.3496	0.5371	2.5170	0.5794	0.8573	2.5731	0.9935
overall	0.2822	0.3623	0.3258	0.8909	3.1569	1.1055	1.5655	3.7979	1.7568

**Table 4:** Parameter effection on classifier accuracy.

Number of Blocks in Each Phase	Feature Dimension of Three-Phase Signals	Optimal Parameter (C, *γ*)	Accuracy (%)
1 × 3	3	(2^6^, 2^5^)	86.14
2 × 3	6	(2^7^, 2^3^)	89.32
4 × 3	12	(2^8^, 2^−2^)	96.82
5 × 3	15	(2^10^, 2^−4^)	98.18
8 × 3	24	(2^8^, 2^−5^)	99.77
10 × 3	30	(2^7^, 2^3^)	99.55
20 × 3	60	(2^8^, 2^−7^)	99.77
40 × 3	120	(2^9^, 2^−7^)	99.77
2000 × 3	6000	(2^8^, 2^3^)	99.55

**Table 5:** Structure parameters of ELM and BPNN.

Architecture of BPNN		Architecture of ELM	
The number of layers	3	The number of layers	3
The number of neuron in the input layer	45 (LE) 36 (EE, SE)	The number of neuron in the input layer	45 (LE) 36 (EE, SE)
The number of neuron in the hidden layer	11 (LE) 10 (EE, SE)	The number of neuron in the hidden layer	14 (LE) 12 (EE, SE)
The number of neuron in the output layer	10	The number of neuron in the output layer	10
The initial weights and biases	Random	The initial weights and biases	Random
Activation	Tansig; Tansig; Logsig	Activation	Sigmoid

**Table 6:** Accuracy of different short-circuit faults classification methods.

Method	Accuracy (%)										Average Accuracy (%)
	AG	BG	CG	ABG	BCG	CAG	AB	BC	CA	ABC/ABCG	
EWT + LE + SVM	100	100	100	100	100	100	100	100	100	98.75	99.77
EWT + SE + SVM	90	87.5	95	82.5	90	92.5	92.5	97.5	95	83.75	90
EWT + EE + SVM	85	87.5	87.5	90	85	82.5	92.5	95	90	91.25	88.86
EWT + LE + ELM	95	92.5	97.5	87.5	90	87.5	97.5	100	100	97.5	94.77
EWT + SE + ELM	67.5	57.5	52.5	27.5	57.5	62.5	90	90	100	73.75	68.41
EWT + EE + ELM	62.5	42.5	50	17.5	37.5	40	65	67.5	70	51.25	50.45
EWT + LE + BPNN	95	97.5	100	100	100	100	100	97.5	100	97.5	98.64
EWT + SE + BPNN	92.5	87.5	85	90	92.5	92.5	95	97.5	90	82.5	89.77
EWT + EE + BPNN	70	82.5	87.5	80	67.5	80	90	75	7.5	76.25	72.05

**Table 7:** Comparison of computing time of different classifiers.

Classifier	Training Time (s)	Testing Time (s)
SVM	46.215	0.149
BPNN	1976.420	0.218
ELM	1.691	0.117

**Table 8:** Classification accuracy of the proposed method with signal adding different noise.

Noise (dB)	Accuracy of Single Fault Type Signals Classification (%)										Overall Accuracy (%)
	AG	BG	CG	ABG	BCG	CAG	AB	BC	CA	ABC/ABCG	
27	97.5	100	95	100	100	97.5	100	100	100	97.50	98.86
33	97.5	100	97.5	100	97.5	100	100	100	100	98.75	99.06
38	97.5	100	100	100	97.5	100	100	100	100	98.75	99.32
43	97.5	100	100	100	100	100	100	100	100	98.75	99.55
57	100	100	100	100	100	100	100	100	100	98.75	99.77

**Table 9:** Actual short-circuit fault detection results.

Fault Type	Fault Detection Time for Every Phase (ms)			FDR (ms)
	A-Phase	B-Phase	C-Phase	
AG	45.2	48.6	45.8	45.2
BCG	45.0	44.8	44.4	44.4
BC	65.2	42.8	42.8	42.8
ABC	45.8	46.4	46.4	46.4

**Table 10:** Actual short-circuit fault classification results.

Fault Type	AG	BG	CG	ABG	BCG	CAG	AB	BC	CA	ABC	Overall Accuracy (%)
AG	32	0	0	0	0	0	0	0	0	0	100
BG	0	22	0	0	0	0	0	0	0	0	100
CG	0	0	27	0	0	0	0	0	0	0	100
ABG	0	0	0	7	0	0	0	0	0	0	100
BCG	0	0	0	0	9	0	0	0	0	0	100
CAG	0	0	0	0	0	11	0	0	0	0	100
AB	0	0	0	0	0	0	12	0	0	0	100
BC	0	0	0	0	0	0	0	18	0	0	100
CA	0	0	0	0	0	0	0	0	16	0	100
ABC	0	0	0	0	0	0	0	0	0	13	100

## Abbreviations

EWT	Empirical Wavelet Transform
WT	Wavelet Transform
WPT	Wavelet Packet Transform
ST	S-Transform
EMD	Empirical Mode Decomposition
EEMD	Ensemble Empirical Mode Decomposition
CEEMDAN	Complementary Ensemble Empirical Mode Decomposition with Adaptive Noise
IMF	Intrinsic Mode Function
NNs	Neural Networks
BPNN	Back-Propagation Neural Network
ELM	Extreme Learning Machine
SVM	Support Vector Machine
MM	Modulus Maxima
LE	Local Energy
EE	Shannon Energy Entropy
SE	Shannon Singular Entropy
FDR	Fault Detection Result
